# A Space Weather Forecasting System with Multiple Satellites Based on a Self-Recognizing Network

**DOI:** 10.3390/s140507974

**Published:** 2014-05-05

**Authors:** Masahiro Tokumitsu, Yoshiteru Ishida

**Affiliations:** 1 Department of Electrical and Control Engineering, Yonago National College of Technology, Hikonacho 4448, Yonago, Tottori 683-0854, Japan; 2 Department of Computer Science and Engineering, Toyohashi University of Technology/1-1, Tempaku, Toyohashi, Aichi 441-8580, Japan; E-Mail: ishida@cs.tut.ac.jp

**Keywords:** self-recognizing network, sensor networks, space weather, *in-situ* sensing

## Abstract

This paper proposes a space weather forecasting system at geostationary orbit for high-energy electron flux (>2 MeV). The forecasting model involves multiple sensors on multiple satellites. The sensors interconnect and evaluate each other to predict future conditions at geostationary orbit. The proposed forecasting model is constructed using a dynamic relational network for sensor diagnosis and event monitoring. The sensors of the proposed model are located at different positions in space. The satellites for solar monitoring equip with monitoring devices for the interplanetary magnetic field and solar wind speed. The satellites orbit near the Earth monitoring high-energy electron flux. We investigate forecasting for typical two examples by comparing the performance of two models with different numbers of sensors. We demonstrate the prediction by the proposed model against coronal mass ejections and a coronal hole. This paper aims to investigate a possibility of space weather forecasting based on the satellite network with *in-situ* sensing.

## 1. Introduction

Satellites provide an important societal infrastructure. They are used for many different applications, including broadcasting, communications, global navigation, positioning, meteorology, Earth observation, science, technology, security, and defense. Satellites are equipped with various sorts of electric devices and instruments for their various missions. However, the operation of satellite instruments is affected by space environmental conditions. High-energy electrons at geostationary orbit (GEO) levels can cause charging of the surfaces of cables and circuits in a spacecraft. Thus, high-energy electrons can penetrate deeply into circuits, and the penetration could lead to deep dielectric charging.

For example, on 20 January 1994 an Intelsat K spacecraft at GEO lost altitude control owing to the failure of the momentum wheel control circuit. According to observations by Geostationary Operational Environmental Satellites (GOES), high-energy electron flux increased greatly during the Intelsat anomalies. Studies on the accident reported that these spacecraft anomalies occurred as a result of dielectric charging by high-intensity and long-duration enhancement of high-energy electrons [1,2]. These studies also reported that spacecraft anomalies at GEO are associated with the enhancement of high-energy electron flux.

Paulikas and Blake [3] found that MeV electron flux at GEO is enhanced 1–2 days after the passage of a high-speed solar wind stream, Turner and Li [4] refer to the variability in the electron flux resulting from geomagnetic storms, which in turn are usually triggered by intense coronal mass ejections (CMEs). Therefore, the enhancement of high-energy electron flux depends on and correlates with both coronal holes and CMEs. Therefore, the prediction of high-energy electron flux caused by these two events is a central challenge in space weather.

The dynamics of high-energy electrons is still under investigation [5], though specialists in space physics have observed the enhancement. Many studies, however, have reported that the enhancement of high-energy electrons is correlated with high-speed solar wind [6,7]. High-energy electron flux is known to be controlled by the solar wind. Furthermore, the north-south component of the interplanetary magnetic field (IMF) is also known to be another important parameter promoting flux enhancement.

Many prediction models for high-energy electron flux at GEO have been proposed. The motivation for developing predictors is to protect spacecraft from deep dielectric charging by providing vital information for operational teams. Methods based on statistical and neural network approaches have been proposed. A linear prediction filter has been developed using a statistical approach [8]. That model is capable of predicting the daily averages of electron flux at GEO. Koons and Goorney have also investigated a predictor of the daily averaged flux at GEO using artificial neural networks [9]. Predictors based on neural network techniques have been developed for high-energy electron flux 24 h ahead [6,10–12].

We propose a novel forecasting system for high-energy electron flux (>2 MeV) at GEO using multiple sensors located at different positions. In a previous study [13], the forecast model incorporates four sensors from two satellites that observe solar wind parameters and high-energy electron flux. This paper extends the model to include multiple sensors monitoring the same kinds of states observed from multiple satellites at different positions. The relations between sensors are estimated using a support vector machine (SVM) [14] for machine learning. We demonstrate the predictions for typical examples, a coronal hole and coronal mass ejections (CMEs).

This paper investigates whether our proposed model is applicable to space forecasting, proposing a system composed of multiple sensors with multiple satellites. The proposed forecasting system incorporates a framework of sensor systems with three factors [15], modeling (identifying relations among sensors), profiling (identifying events and their characteristics), and managing trade-offs (training and tuning). This paper further examines this sensor system's framework by extending it to *in-situ* sensing for space weather forecasting based on sensors from multiple satellites.

In our model, the prediction of space weather is done on the satellite network. Our proposed model assumes that satellites record the observation data and they exchange information of anomaly detection of the sensor data. A related study [16] has investigated the Standard Radiation Environment Monitor (SREM) that provides radiation hazard alarms to the host spacecraft. The SREM units are on-board INTEGRAL and Rosetta of the satellites for monitoring protons. In this study, the demonstration study showed that several SREMs (monitoring different energy bands) units successfully generated alarms of the radiation hazard for the satellites. On the contrary, space weather forecasting also can be done on a computer (*i.e.*, workstation) at Earth. A study [17] investigated a possibility of early warning of radiation hazards by physics-base model. In these circumstances, the two kinds of forecasting manner can be considered in space weather. Our study aims to investigate a possibility of space weather forecasting on the satellite network with *in-situ* sensing and adaptive information processing.

After explaining the data used in Section 2.1, Section 2 then examines modeling (Section 2.2) and profiling (Section 2.3). Managing trade-off is conducted by an SVM in Section 2.2. To extend the framework to *in-situ* sensor systems from multiple satellites, Section 3 compares the performances of the four-sensor (from two satellites) model and the eight-sensor (from four satellites) model. Two typical examples are tested with the proposed model: a coronal hole and CMEs. Section 4 discussed a significance of our proposed model. Furthermore, this paper states remained issues for realizing space weather forecasting system based-on a self-organizing network. Section 5 provides summary and concludes this paper.

## 2. Data and Sensor System

### 2.1. Data

This paper uses three types of data with seven sensors obtained in observations from four satellites. The data types are solar wind speed, north-south component of IMF, and high-energy electron flux. The three of the four satellites observe the solar wind parameters of solar wind speed and north-south component of IMF. These three satellites monitor solar wind parameters at different positions in space. The last data are high-energy electron flux at GEO.

We use one-hour averaged data calculated from observation data of the solar wind parameters and the high-energy electron flux at GEO. Two satellites, STEREO-A and STEREO-B, comprise the Solar Terrestrial Relations Observatory (STEREO) [18]. STEREO-A and B were launched in 2006 into orbits around the Sun that cause them, respectively, to pull further ahead of and fall gradually behind the Earth. The solar wind data observed by STEREO-A and STEREO-B are obtained from the Coordinated Data Analysis Web (CDAWeb) [19] in the National Space Science Data Center (NSSDC) of the National Aeronautics and Space Administration/Goddard Space Flight Center (NASA/GSFC).

The solar wind data observed by the Advanced Composition Explorer (ACE) satellite are obtained from the OMNI-2 database [20] in the National Space Science Data Center (NSSDC) of the National Aeronautics and Space Administration/Goddard Space Flight Center (NASA/GSFC). The electron flux data observed by the GOES satellite are obtained from the National Geophysical Data Center (NGDC) and the National Oceanic and Atmospheric Administration (NOAA/NGDC) [21]. We obtained all data during the period from 2 March 2007 to 7 December 2009, approximately two years in total.

### 2.2. Modeling: Identifying Relations among Sensors

The proposed forecasting system is based on a *dynamic relational network* [22]. The dynamic relational network model is a self-recognition network in which sensor nodes evaluate each other to detect faulty sensors and events of interest. Each sensor node is capable of information processing for manipulating sensor data. The status of each sensor node is represented by credibility. The credibility of a sensor is indicated as a continuous value ranging from 0 (not credible) to 1 (fully credible). An important function of the dynamic relational network is to evaluate the sensor data based on consistency with other sensor data within the sensor system. As the data change dynamically, the consistency also changes, and hence the evaluations as well. Another role of the model is to define and generate *profiles* in several ways. For example, the structure (of the network expressing relations among sensors) and model parameters (such as threshold to determine whether the sensor data are consistent with the relations) are used as profiles.

Dynamic relational networks are used for detection of events in a combustion system of an automobile engine [23] and for home intrusion detection [15]. The dynamic relational network model is also applied to space weather prediction for high-energy electron flux 24 h ahead at GEO [13].

**Figure 1.**
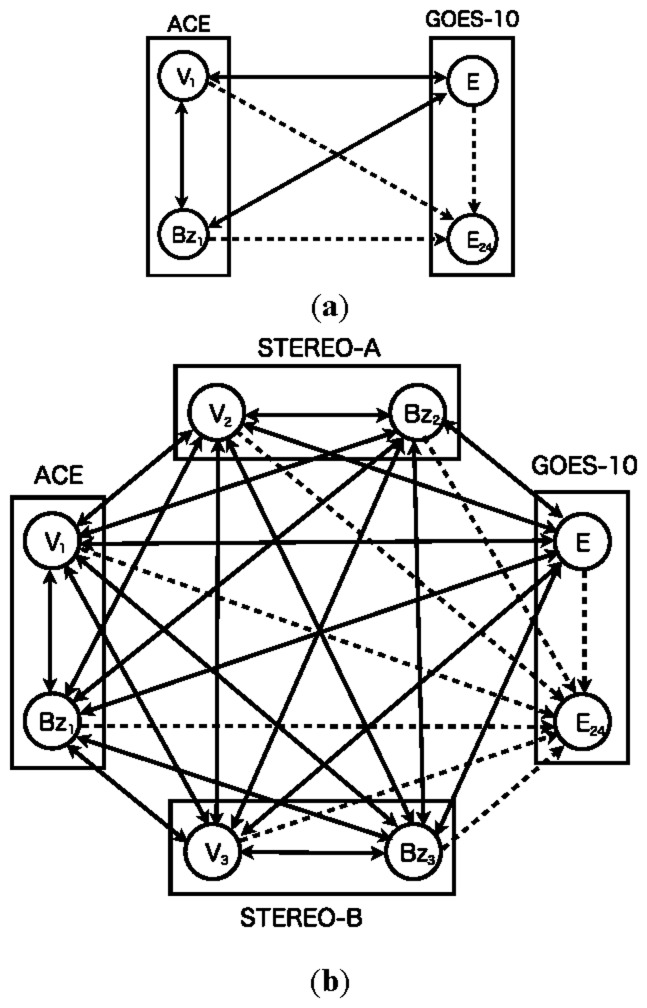
Dynamic relational network for space weather forecast using multiple sensors. (**a**) Four-sensor model (**b**) Eight-sensor model. *V_i_* and *B_i_* (*i* = 1, 2, 3) represent sensor nodes of solar wind speed and north-south component of interplanetary magnetic field. *E* and *E_24_* indicate, respectively, a present high-energy electron flux and its flux 24 h ahead. Rectangles represent regions of satellites equipped with sensors for observation. Solid line arcs represent evaluations from a source node to a target node. Dashed line arcs indicate evaluations involving a prediction from a source node to a target node.

Figure 1 shows both the previous model (Figure 1a) [13] and the proposed model (Figure 1b). The network consists of nodes corresponding to real sensors and a node corresponding to an imaginary sensor. Real sensors are those monitoring solar wind speed, north-south component of interplanetary magnetic field, and high-energy electron flux at GEO. The imaginary sensor represents the high-energy electron flux 24 h ahead. The imaginary sensor is used for prediction of a flux alert level.

The previous model (Figure 1a) involves sensors equipped with two satellites. The network contains three real sensors: a single north-south component of IMF, solar wind speed, and high-energy electron flux. The proposed model (Figure 1b), on the other hand, involves seven sensors in total, three north-south components of IMF, three solar wind speeds, and one high-energy electron flux. Both models include a single imaginary sensor of high-energy electron flux 24 h ahead.

There are three kinds of computation model for a dynamic relational network [22]: *black and white* model, *skeptical* model, and *gray* model. In the *black and white model*, a credibility of a sensor node converges either normal (credibility = 1) or fault (credibility = 0) as a diagnosis proceeds. In the *skeptical model*, a sensor node evaluates a target sensor as normal if only they evaluate normal each other. In the *skeptical model*, therefore, the diagnosis process is more careful than *black and white* model. For the *skeptical* model, however, the convergence tendency of the credibility is same in the *black and white* model. The credibility of the *skeptical* model also converges to the above condition.

We use the gray model for prediction. In the *gray model*, the credibility can be an intermediate value between 0 and 1. The credibility of the *gray model* can reflect ambiguous states in which the status of the sensor normal/abnormal is not determined exactly. The space weather forecasting of high-energy electron flux also involves ambiguous situations in which the change of the flux is unpredictable. Therefore, the gray model is suitable for forecasting of high-energy electron flux in this study.

A warning to alarm is determined by comparing with the threshold and the credibility of the sensors in operation. The alarm thresholds for warning can be tunable parameters, because the threshold is controlled by a policy of spacecraft operational teams. For instance, the alarm threshold can be smaller value less than 0.5. For this case, this policy selects safety operation since the sensor sensitivity for anomaly detection becomes sensitive. The alarm threshold is 0.5 and fixed value in this paper.

The source node evaluates the target as abnormal, if the relation between two sensors deviates from a predefined condition. For instance, if the relationship between solar wind speed and high-energy electron flux deviates from the predefined condition, then the source node evaluates the target node as abnormal.

We use *R_i_* to denote the credibility of sensor node *i*. The credibility is a time variable, so the value will be changed by mutual evaluation according to time development. We denote the relationship between two sensors as *T_ij_*. The value of the parameter *T_ij_* is determined by the diagnosis from the source node *i* to the target node *j*. The source node *i* evaluates the target node *j* based on their relation. The relation is expressed as an arc in the network to be constructed using SVM. The dynamics of the dynamic relational network are described as follows [22]:

(1) $$\frac{dr_{i}(t)}{dt}=\sum_{j}T_{ji}^{+}R_{j}(t)-r_{i}(t)$$

where:

(2) $$R_{i}(t)=\frac{1}{1+\exp(-r_{i}(t))}$$

(3) $${T^{+}}_{ij}=\{\begin{array}{ll} T_{ij}+T_{ji}-1 & \text{if there are arcs between}\;i\;\text{and}\;j\\ 0 & \text{if there is no arc between}\;i\;\text{and}\;j \end{array}$$

*r_i_*(*t*)∈( −∞, +∞) is a time variable which represents an accumulation of a diagnosis result. Equation (1) describes the dynamics of the credibility for a sensor node *i*. Equation (2) defines a mapping function of *r_i_*(*t*) to *R_i_* (*t*) ∈ [0,1]. The time variable *r_i_*(*t*) is translated into the credibility *R_i_* through Equation (2).

The relation between two sensors is estimated using an SVM. The parameters of the SVM represent the relation between two sensors as a profile. In the previous model [13], the relations were estimated by Vector Autoregressive Models (VAR models). The space environment data, the interplanetary magnetic field in particular, changes rapidly in a short duration, thereby requiring statistical parameters for some duration to detect the change. The VAR models are not sufficient for prediction because they do not follow chaotic changes of the observed values. The north-south component of IMF changes irregularly between south and north directions. For our purpose, the VAR models could not predict the values from time-series data.

We could not incorporate the statistical parameters in VAR models, but they can be involved in the SVM. The SVM is an outstanding method in machine learning for classifying input data into two categories: +1 and −1. The status of normal and fault for the sensors correspond to two categories +1 and −1. The SVM evaluates the relation between two sensors appropriately so that the SVM classifies the input data nonlinearly into two categories. Therefore, the SVM is selected as a profiling method for estimating relations between two sensors.

For applying the SVM to space weather forecasting, the training and test process are necessary for consideration. The input parameters and desired output are determined for training process. For the test process, the input data is passed to the SVM and its outputs the class normal/fault. The appropriate selection of the input parameters for the SVM is crucial in order to bring out its classification performance. This paper uses time-averaged values as the input parameters instead of simple time-series data, which represent feature for some duration of the observed data.

The relation between two (sensor) nodes *i*, *j* is learned using the four types of data, three days average and standard deviation of the two sensor data. The parameter *T_ij_* is determined by evaluating the relation between the two nodes based on the profiles generated by the SVM. If any of the four types of data, e.g., standard deviation of the sensor node *j*, deviates, then the relation from node *i* to node *j* is *T_ji_* = −1; otherwise *T_ji_* = 1. For prediction purposes, an imaginary sensor node *E*_24_ represents a high-energy electron flux 24 h ahead. We have two types of data, normal data without the event and abnormal data with the event having occurred. The normal data contain the event in which the high-energy electron flux possibly keeps a safety level 24 h ahead. The relation from a node to the node *E*_24_ is trained to give −1 when the event occurred after 24 h and +1 when the event did not occur after 24 h. There can be no arc from the imaginary sensor node *E*_24_.

### 2.3. Profiling: Identifying Events and Their Characteristics

Since the mission of sensor systems is detection of events, we define profiles as information that can be used to detect events. Profiles may be structured hierarchically from specific events to an intermediate category of collections of events, or events up to the most general kind of abnormal event (and its opposite, the normal event).

**Figure 2.**
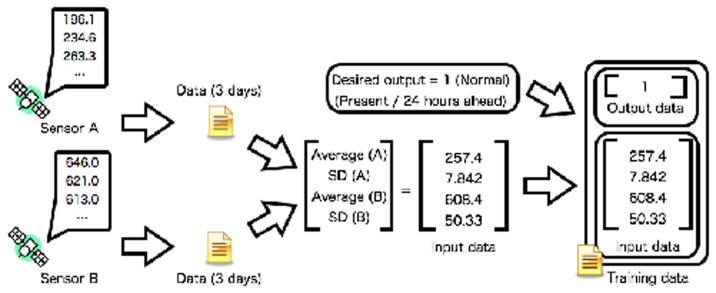
Training data generation process. Training data are created from observed data of two sensors on satellites. The training data consists of averaged values, the standard deviations, and the desired output.

Profiles are created from statistical data using an SVM [14]: the time-averaged value for the past three days and its standard deviation for each sensor (Figure 2). Both the time-averaged value and its standard deviation are sensitive to the event of interest. For instance, high-speed solar wind is observed during an appearance of a coronal hole of the Sun. In this situation, the time-averaged value increases rapidly and its standard deviation is large. The quiet condition (low-speed solar wind) is quite different from the condition during observation of high-speed solar wind. The event of high-energy electron flux can be considered similarly.

The profile is described as the parameters of the SVM. The parameters of the SVM are generated from training dataset. The input data for training is created from two kinds of time-series data recorded by the sensors on the satellites. Each time-series data represents the hourly time-averaged values. The time-averaged values and their standard deviations are calculated from the time sequences, and then the input data for training is represented as a vector consisting of four values. The desired output expresses present high-energy electron flux or 24 h ahead also is determined. The normal and abnormal conditions of high-energy electron flux are determined by space weather criteria [24]. The space condition is usually regarded as safe when the high-energy electron flux is smaller than 10^3^ and at the warning level otherwise. For training data generation process, the training data are finally determined with the desired output and input data. The training data for a relation between two sensor nodes consist of four values: time-averaged values for the past three days and their standard deviations for each type of sensor data.

**Figure 3.**
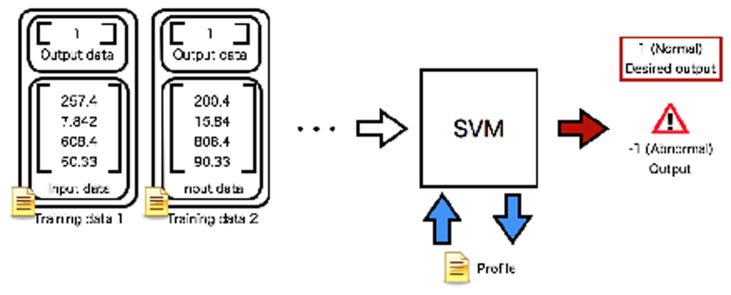
Training process of a profile between two sensors. The training data are input to an SVM. The SVM learns desired outputs from the training data set.

The profiles are respectively generated for a tuple of the two sensors (Figure 3). Figure 3 shows an example in that the learning process creates the relation between the desired output and the input training data. The training dataset is prepared and used for learning of the parameters for the SVM. In the learning process, the parameters of the SVM are optimized to make the relation between the input data and desired output. The optimized parameters obtained from the learning process are saved as the profile.

**Figure 4.**
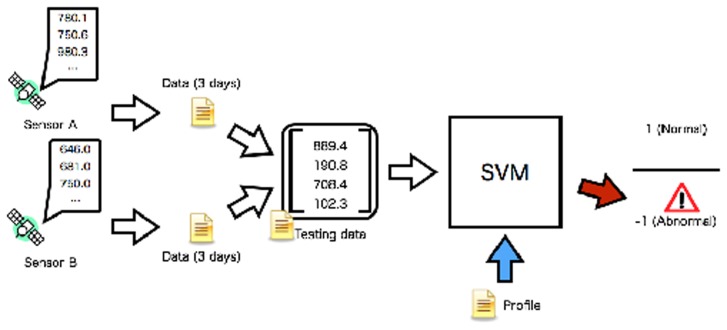
Test of observed data recorded by two sensors. The test data are input to an SVM based on the profile. The SVM classifies the condition of the space environment as binary values 1 (normal) or −1 (abnormal).

The dynamic relational network for detecting the events called *appearance of a coronal hole at the Sun* and CMEs are considered, and the observed data are given to the sensor system. Six sensors from three satellites monitor the solar wind parameters. These sensors monitor the same kind of physical values; however, the spatiotemporal pattern is different owing to their distinct locations. The relation between the two sensors is tested based on the generated profile (Figure 4). The two sensors record the observed data, which are used for the test data for the SVM. The sensor A/B act as a source node and another one does as the target node. In order to create the test data, the time-averaged and its standard deviation are calculated for each sensor. The SVM evaluates the relation between the two sensors based on the test data. The SVM outputs the binary values +1/−1 for the target sensor as the diagnosis result. The output range of the SVM corresponds to values of the parameter Tij. Finally, this test process assigns +1 or −1 to the parameter Tij.

**Figure 5.**
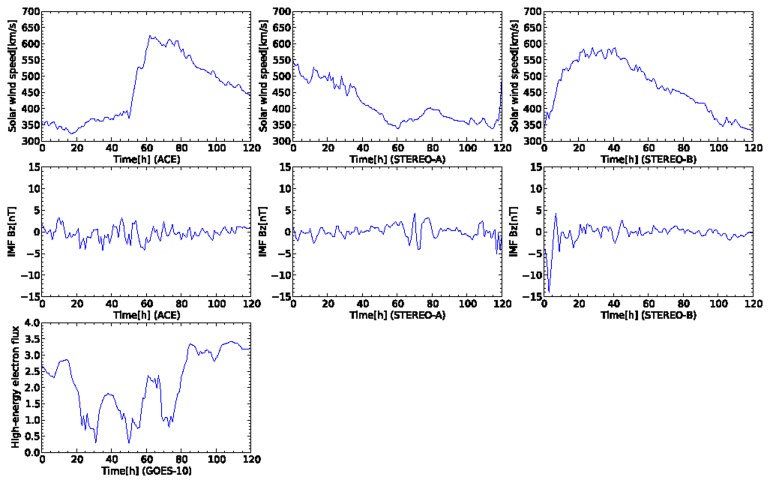
Time development of sensor data for five days from 16 August 2008 at 00:00:00 UTC. This plot contains an event of high-speed solar wind flowing from a coronal hole. In the first and second rows, the plots of the columns correspond to the satellites of ACE, STEREO-A, and STEREO-B, respectively. In the third row, the plot corresponds to GOES-10.

Figure 5 shows plots of an appearance of a coronal hole from 21 August 2008. All the plots were created from 16 August 2008 at 00:00:00 UTC to 21 August 2008 at 23:00:00 UTC. High-speed solar wind is observed at the beginning of the data at STEREO-A. Subsequently, high-speed solar wind data also are observed by the two satellites of ACE and STEREO-B. The solar wind speed observed by ACE rapidly increases from 400 km/s to 600 km/s at the time 52 h from the start of the sensor data. Simultaneously, the north-south component of IMF observed by ACE and STEREO-A also changes rapidly when the high-speed solar wind is recorded. In the plot, the high-energy election flux observed by GOES-10 approaches a warning level at the time 80–120 h after the oscillated north-south component of IMF is observed by ACE. The crucial task for the proposed model is prediction of the warning level of the high-energy electron flux during 80–120 h in the plot.

**Figure 6.**
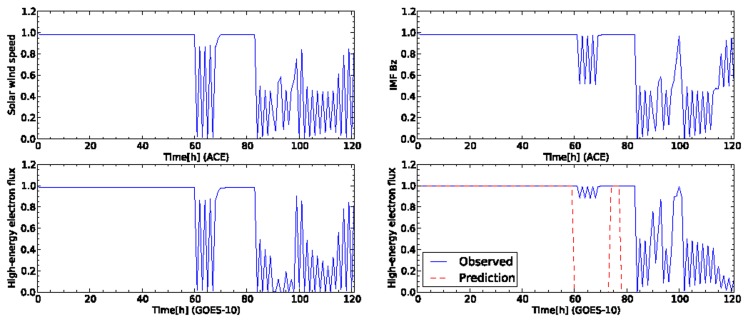
Time development of credibility for the test data shown in Figure 4. The four-sensor model evaluates the test data. The value of a vertical axis represent a credibility corresponding to a sensor name indicated at the vertical axis. The name of the satellite for the sensor is represented at horizontal axis.

The observed data shown in Figure 5 are sent to sensor nodes in the dynamic relational network. Each node reads the sensor data at every step and evaluates the credibility of the target node based on the sign of the arc. The state of the network changes according to the time development of the sensor data. Figure 6 shows the time development of the credibility of each node for the network shown in Figure 1a. Each node keeps high credibility until the high-energy electron flux increases at the time 60 h from the start of the data. The network detects the enhancement of the high-energy electron flux. However, the credibility of each sensor is unstable. The high-energy electron flux 24 h ahead detects the increase of flux after 80 h from the start of the data. The credibility of the high-energy electron flux 24 h ahead is still unstable. The node of the high-energy electron flux outputs an ambiguous prediction result. The credibility for the high-energy electron flux oscillates dynamically. Other nodes also report ambiguous results by detecting a deviation in the usual observation data.

**Figure 7.**
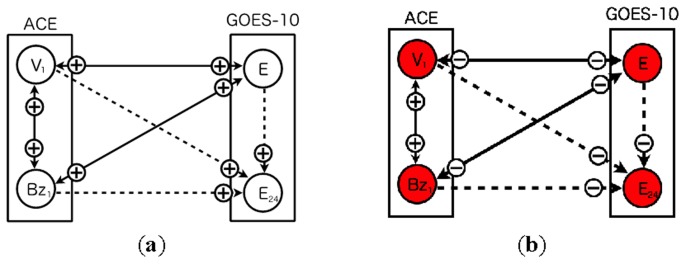
Snapshot of nodes in the dynamic relational network (four-sensor model). The nodes evaluated as faulty from all sensors are colored red. The nodes evaluated as faulty from half nodes are also colored red. The plus and minus signs on arcs represent a diagnosis result (plus = normal/minus = abnormal). Rectangles represent regions of satellites equipped with sensors for observation. The plus and minus signs on arcs represent a diagnosis result (plus = normal/minus = abnormal). The solid lines indicate two nodes diagnose each other. The dashed lines indicate unidirectional diagnosis from one node to another one. The symbol ***V****_1_* and ***B****_z1_* respectively represent solar wind speed and north-south component of interplanetary magnetic field. The symbol ***E*** and ***E****_24_* respectively represent high-energy electron flux and its 24 h ahead. (**a**) Initial state (Step = 0); (**b**) Step = 84.

Figure 7 shows snapshots of the network based on the four sensors from two satellites. At the initial step, all nodes are evaluated as normal (Figure 7a). The plus and minus symbols on arcs represent evaluation: normal or abnormal. The criterion for abnormal detection of sensors is determined by comparing with a credibility and warning threshold. The warning threshold is 0.5 in this paper. The sensor node can be colored red if the credibility of the sensors exceeds the threshold. The symbols on arcs change if the sensor data deviates from the predefined conditions. The rectangle indicates a satellite with which a sensor is equipped.

Figure 7b shows a sensor status of each sensor in the network at step 84. The status of the sensors is decorated by color according to its credibility. The diagnosis result of the arcs also is shown whole the network. At this step, the two nodes of the high-energy electron flux are evaluated as abnormal. Furthermore, other nodes of the north-south component of IMF and the solar wind speed are also evaluated as abnormal. These nodes are colored red where the credibility is very small, about 0.2. In this simulation, the credibility of the nodes oscillates between two states, 0 (not credible) and 1 (fully credible).

## 3. Event Monitoring by Sensor Systems for Multiple Satellites

### 3.1. Case Study 1: Enhancement of High-Energy Electron Flux by a Coronal Hole

**Figure 8.**
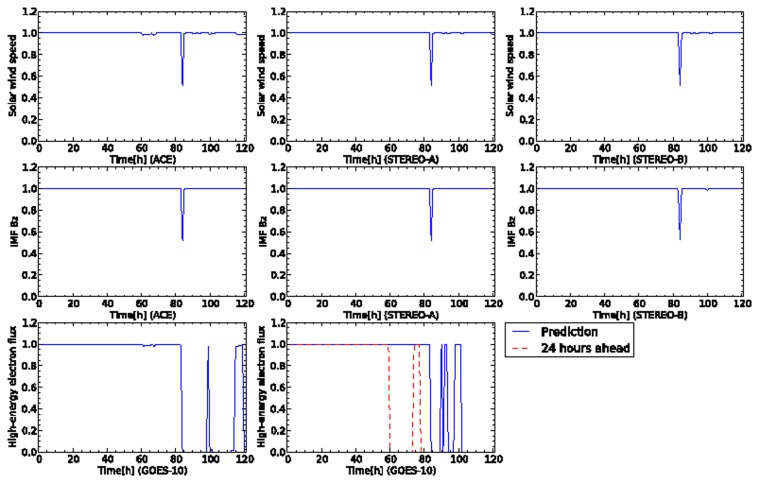
Time development of credibility for the data shown in Figure 4. The eight-sensor model evaluates the test data. The value on a vertical axis represents a credibility corresponding to a sensor name indicated on the vertical axis. The name of the satellite for the sensor is represented at horizontal axis. In the first and second rows, the plots of the columns correspond to the sensors on ACE, STEREO-A, and STEREO-B, respectively. In the third row, the left-most and right-most plots correspond, respectively, to GOES-10 and GOES-10 24 h ahead.

**Figure 9.**
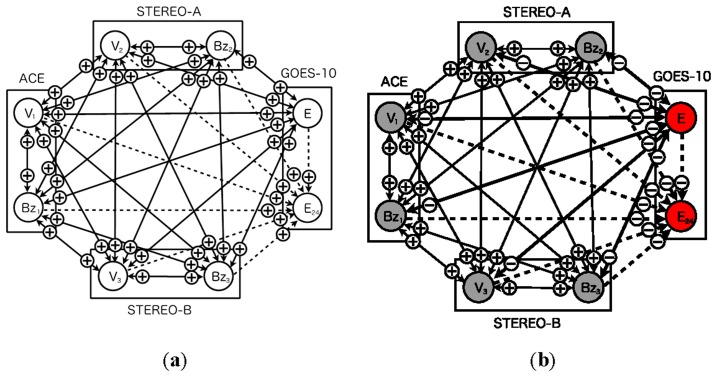
Snapshot of nodes in the dynamic relational network (eight-sensor model). The nodes evaluated as faulty from all sensors are colored red. The nodes evaluated as faulty from half nodes are also colored red. The plus and minus signs on arcs represent a diagnosis result (plus = normal/minus = abnormal). Rectangles represent regions of satellites equipped with sensors for observation. The solid lines indicate two nodes diagnose each other. The dashed lines indicate unidirectional diagnosis from one node to another one. The symbol ***V****_i_* and ***B****_zi_* (*i* = 1, 2, 3) respectively represent solar wind speed and north-south component of interplanetary magnetic field. The symbol ***E*** and ***E****_24_* respectively represent high-energy electron flux and its 24 h ahead. (**a**) Initial state (Step = 0); (**b**) Step = 84.

We compare the performance of the forecasting systems based on the four-sensor (from two satellites) and eight-sensor (from four satellites) models. Figure 8 shows the time development of the credibility predicted by the network based on the eight sensors from the four satellites. Figure 9 shows the snapshot of the nodes in the dynamic relational network at the initial snapshot (Figure 9a) and at step 84 (Figure 9b). Each node outputs high credibility until step 84. These sensors are evaluated as normal. This network also fails to detect a warning level of the high-energy electron flux between steps 60 and 80. At step 84, the credibility of all the nodes decreases to half except the two sensors on high-energy electron fluxes. The credibility of the high-energy electron flux is evaluated as entirely abnormal at this step. After this step, all nodes retain high credibility except the high-energy electron flux. The high-energy electron flux 24 h ahead shows a little change in the credibility between steps 80 and 100. Subsequently, however, the high-energy electron flux 24 h ahead shows low credibility, because it predicts the warning condition of high-energy electrons at GEO. The eight-sensor model by four satellites is successful for prediction of warning level of the flux. Furthermore, the credibility of the flux 24 h ahead does not lead to the oscillation observed in the network by the four-sensor model.

### 3.2. Case Study 2: Rapid Decrease of High-Energy Electron Flux by a Coronal Mass Ejection

**Figure 10.**
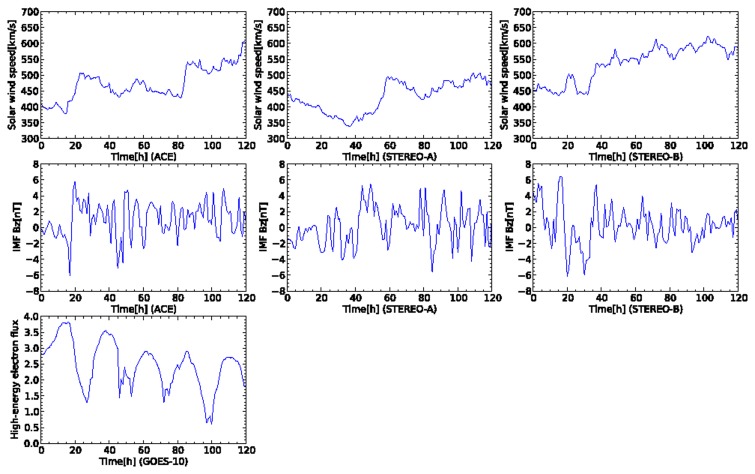
Time development of sensor data for five days from 30 April 2008 at 00:00:00 UTC. This plot contains an event of high-speed solar wind flowing from a coronal hole. In the first and second rows, the plots of the columns correspond to the satellites of ACE, STEREO-A, and STEREO-B, respectively. In the third row, the plot corresponds to GOES-10.

Another crucial space weather event is CMEs. Next, we demonstrate the prediction for CMEs by our proposed model. Figure 10 shows the time development of sensor data for the CME happened at 26 April 2008. All the plots were created from 30 April 2008 at 00:00:00 UTC to 4 May 2008 at 23:00:00 UTC. In this event, several days later, the solar wind is ejected in front of the Earth from the surface of the Sun. The rapid increase of the solar wind speed was observed by ACE at the time 20 h from the beginning of the sensor data. Simultaneously, the north-south component of IMF also rapid decreased to the south direction. Other observation data for the north-south component of IMF and solar wind speed for STEREO-A and TEREO-B showed monthly variation originated from a coronal hole. The high-energy electron flux usually rapid decreases a few days later when the rapid increase of solar wind speed is observed by ACE. The high-energy electron flux shows the smallest value at the time 100 h from the start of the sensor data. Then, the high-energy electron flux rapidly increases and keeps its level several days.

**Figure 11.**
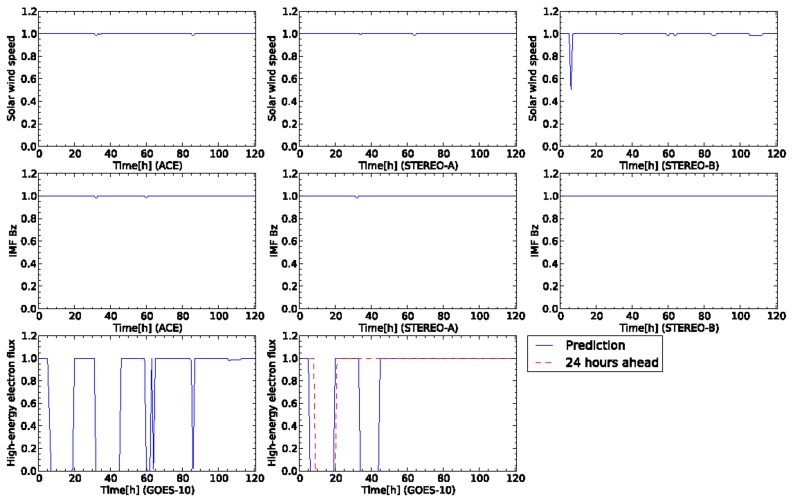
Time development of sensor data for five days from 30 April 2008 at 00:00:00 UTC. This plot contains an event of high-speed solar wind flowing from a coronal hole. In the first and second rows, the plots of the columns correspond to the satellites of ACE, STEREO-A, and STEREO-B, respectively. In the third row, the plot corresponds to GOES-10.

Figure 11 shows the time development of the credibility of each sensor. The test data is shown in Figure 10. The two sensors for solar wind speed of ACE and STEREO-A indicate high credibility for the whole of the sensor data. The solar wind speed of STEREO-B, however, shows the rapid variation of the credibility in the beginning of the sensor data. This is induced by the sudden changes of the solar wind speed and north-south component observed by STEREO-B at the time a day ago.

For the high-energy electron flux of GOES-10, the sensor node is diagnosed as abnormal because the credibility shows 0.0 in short duration several times. This diagnosis result corresponds to the changes of the high-energy electron flux observed by GOES-10. For the sensor of the high-energy electron flux 24 h ahead, the credibility follows the observed flux except the duration from about 35 to 45 h. The proposed model also predicts the decrease of the high-energy electron flux in the recovery phase.

In the eight-sensor model, the deviations in the predefined conditions are detected as a result of the many relations among sensor nodes. These detections contribute to preventing oscillation in sensor credibility. The predictions of the high-energy electron flux 24 h ahead by the eight-sensor model are more reliable than those by the four-sensor model. Solar activity can be observed with higher accuracy by sensors located at distinct locations. Having multiple sensors with different satellites plays a significant role in prediction of the high-energy electron flux 24 h ahead.

## 4. Discussion

Space weather forecasting usually involves a substantial amount of data observed by multiple satellites. In a satellite observation for space weather forecasting, transmission of the observed data to the ground is a critical problem because of the bandwidth for transmitting observed data is limited. The proposed model resolves the transmission bandwidth of observed data in space weather by transferring the data between satellites instead of sending data to ground stations. More importantly, using sensor systems involving sensor data from multiple satellites (hence from distinct positions) allows intelligent *in-situ* sensing by rearranging positions and directions of sensors.

The two satellites of STEREO-A and STEREO-B orbit and monitor the Sun from various positions. Their positions are symmetrical relative to the Earth, following an elliptic curve. The two satellites detect the distinct solar wind parameter because the solar wind is expanded spirally owing to rotation of the Sun. The proposed forecasting system takes advantage of spatiotemporally distinct solar wind parameters.

The proposed forecasting system allows the prediction of high-energy electron flux involving more than three sensors. The proposed model successfully forecasts the high-energy electron flux 24 h ahead. Whereas mutual evaluation with only two sensors leads to an oscillation in the credibility when the high-energy electron flux approaches the warning level, evaluation with more than three sensors prevents credibility fluctuation. The three satellites in the proposed model observe the solar wind speed and interplanetary magnetic field. The credibility of the high-energy electron flux converges to zero in this evaluation, leading to accurate forecasting for typical examples of a coronal hole and CMEs.

The proposed model forecasts the binary status of whether the flux level is higher than the threshold. The forecast is conducted by evaluating the credibility of the sensors. The credibility is calculated by evaluating the data obtained from multiple sensors observing the same kinds of data but at distinct locations. The observation is done spatiotemporally from multiple satellites at distinct locations. The previous model involves only two sensors: a solar wind parameter and a high-energy electron flux [13]. The proposed model involves multiple sensors located at distinct locations for forecasting, thereby allowing more reliable forecasting than that of the previous model.

Our proposed model is different approach compared with ones such as statistical analysis [2], neural networks [6,10–13] and physics-based model [25]. The proposed model expands *in-situ* sensing that not only monitors space environmental data but also processes (evaluates) observed data. In the statistical analysis and neural networks, the prediction of space weather can be done on a computer. These two approaches need to collect the observed data from the satellites into ground stations of one space. In our proposed model, sensors on the satellites exchange the observed data and they diagnose each other based on their predefined profiles. The proposed model suggests the way for space weather forecasting without transferring huge data to ground stations. The demonstrations, in this paper, suggest that the space weather forecasting based on the satellite network would be capable in real-time in space.

We are able to extend the dynamic relational network by adding links between sensors of the satellites in the proposed model. The proposed model is coped with a new arrangement of a satellite for the satellite network. The relations between two sensors can be described by several ways such as neural networks, statistical analysis and hidden markov models [15]. Our proposed model involves new findings on space physics to the prediction. For instance, a correlation between two observed data is revealed, this achievement is applicable to the prediction model by generating profiles. The physics-based fact is also applicable to the our proposed model, if the fact is available between the two kinds of quantitate values.

Furthermore, the proposed model is adaptive for changing a structure of the satellite network. The satellite network for the prediction is robust when one satellite leaves from the satellite network because the two satellites only change their links. The satellites need to change their locations according to their mission progresses. Rewiring between the two satellites can change the logical structure of the satellite network. Therefore, our proposed model is suitable for space weather forecasting with *in-situ* sensing.

Two issues must be discussed in order to apply the proposed model to satellite networks. The first issue is the location of the satellites making up the satellite network for the space weather forecasting. The communication for exchanging data between satellites would be difficult due to their distance or location. For this case, an arrangement of simple transponders for relaying the observed data is addressed or observation satellites transfer the data for the destination of the satellite. The storage space of the observation satellites is extremely limited due to their purpose. The transponders also provide storage space for the observation satellites. The observation satellites push or pull the data depending on their demand for use.

The second issue is coverage of the observation. The ACE, STEREO-A and STEREO-B record space environmental data and transfer them to the ground stations. However, the ground stations do not necessarily receive all data submitted by the observation satellites. The operation for the observation also would be suspended due to some technical or managing reasons. In this case, the interpolation of the observed data by other satellites addresses the missing data. The new techniques for processing the observed data are also need to be considered for automation of space weather forecasting.

## 5. Conclusions

A space weather forecasting system was proposed for high-energy electron flux 24 h ahead at GEO. The forecasting was based on multiple sensors from multiple satellites located at different positions. The system consists of seven sensors equipped with four satellites and one imaginary sensor, a sensor for prediction. The proposed model successfully predicts high-energy electron flux using more than three sensors from four satellites; however, prediction using mutually evaluation of two sensors from two satellites leads to an ambiguous forecast. Thus, more sensors are required for *in-situ* sensing to give a more accurate forecast. Further, *in-situ* sensor systems should be organized in terms of sensors from different satellites forming a network for more accurate and reliable forecasting.
